# A low-cost spectroscopic nutrient management system for Microscale Smart Hydroponic system

**DOI:** 10.1371/journal.pone.0302638

**Published:** 2024-05-08

**Authors:** Joseph D. Stevens, David Murray, Dean Diepeveen, Danny Toohey

**Affiliations:** 1 School of Information Technology, Murdoch University, Murdoch, Western Australia, Australia; 2 School of Agricultural Sciences, Murdoch University, Murdoch, Western Australia, Australia; 3 Department of Primary Industries and Regional Development, South Perth, Western Australia, Australia; 4 School of Management and Marketing, Curtin University, Perth, Western Australia, Australia; University of California Davis, UNITED STATES

## Abstract

Hydroponics offers a promising approach to help alleviate pressure on food security for urban residents. It requires minimal space and uses less resources, but management can be complex. Microscale Smart Hydroponics (MSH) systems leverage IoT systems to simplify hydroponics management for home users. Previous work in nutrient management has produced systems that use expensive sensing methods or utilized lower cost methods at the expense of accuracy. This study presents a novel inexpensive nutrient management system for MSH applications that utilises a novel waterproofed, IoT spectroscopy sensor (AS7265x) in a transflective application. The sensor is submerged in a hydroponic solution to monitor the nutrients and MSH system predicts the of nutrients in the hydroponic solution and recommends an adjustment quantity in mL. A three-phase model building process was carried out resulting in significant MLR models for predicting the mL, with an R^2^ of 0.997. An experiment evaluated the system’s performance using the trained models with a 30-day grow of lettuce in a real-world setting, comparing the results of the management system to a control group. The sensor system successfully adjusted and maintained nutrient levels, resulting in plant growth that outperformed the control group. The results of the models in actual deployment showed a strong, significant correlation of 0.77 with the traditional method of measuring the electrical conductivity of nutrients. This novel nutrient management system has the potential to transform the way nutrients are monitored in hydroponics. By simplifying nutrient management, this system can encourage the adoption of hydroponics, contributing to food security and environmental sustainability.

## 1. Introduction

Hydroponics, the soilless technique of growing plants with nutrient infused water, offers flexible configurations that can be customized to space limitations of urban areas to grow food. This technique uses up to 80% less water than conventional farming and grows plants up to three times faster [1–3]. Considering the recent COVID-19 pandemic, where the world witnessed a breakdown of global food supply chains, there is value in exploring ways to empower residents in urban areas to cultivate some of their own food [4]. The management of nutrients in water requires specialized knowledge, and equipment, and is one of the most complex aspects for home hydroponics users, impeding adoption [5]. Nutrients in the hydroponic nutrient solution are depleted as the plant grows and, users must add nutrients periodically. Knowing when, how much, and what nutrients to add is crucial for success. Microscale Smart Hydroponics Systems (MSHS) leverage Internet of Things (IoT) technology and cheap commodity computing components to aid users in hydroponic management [6–8]. However, there is a gap in the area of effective nutrient management in MSHS, that inhibits the adoption of hydroponics for home users [9,10].

This study is to present the development and testing of a novel, low-cost nutrient management system, aimed at reducing the barriers of hydroponics for the home user. An inexpensive IoT spectroscopic sensor, applied in situ, is used to monitor, and recommend nutrient to be added in a metric easy for users to understand. The system performance is then tested in a real-world grow that compares the performance of the nutrient management system to a control group with no nutrient management.

The predominant trend in smart hydroponic nutrient management, uses an analog electrical conductivity (EC) probe [11,12]. The probe administers a small electrical current between a cathode and an anode to determine the total nutrient salt content of a solution by measuring the conductivity. These probes easily integrate with consumer grade IoT systems to manage nutrient content [12]. There has been substantial work in nutrient management systems for MSHS application using the standard EC probe approach [13–27].

The previous research demonstrates that EC probes possess certain qualities that make them suboptimal for MSHS deployment. Firstly, EC probes have a short life span and cannot be left in the solution indefinitely [24,28]. Separate measuring tanks [22,25] or pumping the solution through a pipe with mounted sensors [26] have been proposed but do not address the durability concerns of EC probes. Secondly, because EC probes administer small electrical currents in the water, they are prone to interference from other water sensors that may also use similar techniques. Lastly, there is no way to measure the individual nutrients using only an EC probe, only the overall nutrient content [10]. Considering these limitations, inherent in their design, it is necessary to examine the work done in large scale smart hydroponic nutrient management.

Research in nutrient management of smart hydroponics systems aimed at large scale deployment utilize alternative sensors for nutrient management systems. The two most important innovations in this space have come from the application of Ion Selective Electrodes (ISE) and inline or in situ spectroscopy. ISEs emerged into the hydroponic research space in 2007 [20] and were further developed in later studies [29,30]. These sensors build on traditional EC probes, by placing a thin selective barrier allowing only specific nutrient ions to permeate and reach the EC probe. The first major study where ISE’s were utilized for a nutrient management system in a real world application was reported in 2014 [31], and used a commercially available ISE for nitrate nitrogen (NO3), potassium (K), and calcium (Ca), each costing €400 at the time. The study used distilled water and the nutrient was completely replaced every 60 days. The experiment grew tomatoes from seedling to fruit using the nutrient management system to manage the grow for the last three months. The system took readings and made adjustments every two to five days. The system, on its own, was not able to sufficiently adjust the nutrients and manual addition of nutrients had to be supplemented periodically, but the system was effective at monitoring and identifying nutrient concentrations changes [31].

Jung, Kim [32] applied a similar technique to manage the nutrient solution for a 14-day hydroponic grow lettuce. The system tested its ability to control the nutrients effectively in a real-world grow [32]. The study focused on two custom-made ISE probes to monitor and automate the adjustment of NO3 and K. They used two-week-old lettuce seedlings and transplanted them into the system for the experiment. Adjustments were made every three days to avoid nutrient overages. The experiment was successful, and the system was demonstrated to effectively control NO3 and K [32]. However, there was no control group, and the analysis of the plant growth wasn’t conducted. It should be noted that despite the efforts to limit adding excess nutrients they found that there were slight overages in K.

Belhekar, Thakare [33] employed an onsite method of measuring nitrogen (N), K, and Ca using custom built ISE probes. The system was designed for greenhouse management and the performance of the system was measured through a real-world application of growing one year old paprika over 16 days. The experiment used a standard two-part hydroponic nutrient solution. Nutrient A, containing the N and Ca, and Nutrient B solution containing K. The nutrients were added according to the ISE readings respectively when the levels fell below the targets. Test samples of the solution were analysed in a lab and compared against the performance of the system measurement. The system was able to accurately predict and adjust the N with high levels of accuracy, however there were discrepancies noted for the K and Ca. Over the16-day period one of the measurement chambers became clogged with algae and negatively affected the readings, the system was still able to perform in real-world applications.

As demonstrated in the literature, ISEs offer a promising alternative to standard EC measurements, however, they require further development before they can be considered for deployment in MSHS nutrient management systems. They are costly, delicate and require careful system design to ensure their functionality [31–33].

An interesting development that offers an alternative to ISE’s is applied, inline or in situ spectroscopy. This has yet to be tested in an actual grow demonstration, however, a number of systems have been constructed with this alternative technology. Jung, Kim [34] used a combination of ISE and an offline analysis of prepared samples combined with PCR and PLSR to build a model that could accurately predict the amount of phosphate in a hydroponic solution. However, this offline application of spectroscopy is impractical in a real-world setting of a nutrient management system.

There have been recent interesting developments in inline or in situ spectroscopy application. Silva, Löfkvist [35] proposed a system that pumps nutrient solution through a tube with a penetrative UV-Vis spectrometer (STS-UV-L-50-400, Ocean Insight**)** set up. Using a self-learning AI model, the system was able to successfully observe changes in the N, phosphorus (P) and K. This system, using the self-learning AI model, was then used to test samples that were collected from different stages of growth from a greenhouse [24]. These samples were put through the system and their nutrient content was successfully analyzed showing that this technique could be used to manage a nutrient solution accurately.

The primary is cost. Silva, Löfkvist [35] and de Camargo, Spanhol [24] use spectrometers (Ocean Insight Inc.) that cost hundreds of dollars and are aimed at large scale greenhouse style deployment. Additionally, the system pumps nutrient through a measuring chamber, adding complexity to the system design. To address the shortcomings of these systems for application in a MSH system Stevens, Murray [10] used a variation on the approach instead opting for an inexpensive commodity grade IoT spectroscopy sensors and applying the sensor in the nutrient tank instead of using a sample chamber.

Stevens, Murray [10] presented an IoT spectroscopic system designed for MSH systems, that was able to successfully measure the concentration of N in a hydroponic MSH system using low-cost commodity spectroscopic sensor. This is interesting because even though [24], Silva, Löfkvist [35] were able to detect all three of the major macro nutrients with a lab grade spectrometer applied inline, it was not deployed in a real-world application. Stevens, Murray [10] were able to monitor N concentrations during a real-world 28-day grow of lettuce. The sensor was mounted at the bottom of the nutrient tank and used a SparkFun AS7265x triad spectroscopic sensor and a transflective aluminium mirror, addressing the trouble measuring low nutrient concentrations issue identified by Jung, Kim [34]. The system was able to successfully predict the levels of N concentration in a nutrient solution when compared against the lab analysis for a 40 Litre and 80 Litre tank. However, the acrylic box sensor was mounted in began leaking at the end of the experiment. The work described in Stevens, Murray [10] forms the basis for this work, which shows that it is possible to monitor nutrients with a cheap IoT spectroscopic sensor. There is a clear gap for the development of low cost spectroscopic nutrient management for MSH systems.

## 2. Materials and methods

### 2.1 Sensor housing design and functionality testing

The sensor selection and system build is based on previous work in a similar application [10]. Six SparkFun Triad Spectroscopy Sensor-AS7265x were procured at a cost of $70 USD each. Two were used for testing selection of waterproofing material and four were used for building the system. The AS7265x utilizes three photodiodes that cover the range from 410 nm to 940 nm with 18, 30 nm spectral channels. The board has three LED lights mounted next to their respective respondent photodiodes, a white Luxeon 3014 – L130-57800 LED that peaks at 500 nm, an Everlight SIR19-21C/TR8 that peaks at 875nm and a Vishay VLMU3100-GS08 that peaks at 405 nm. The sensor comes calibrated from the manufacturer. The LEDs flash and the photodiodes detect the reflected light intensity. The AS7265x was selected based on its proven accuracy [7,10,36–38] and ease of system integration in similar applications [10,37].

**Fig 1:**
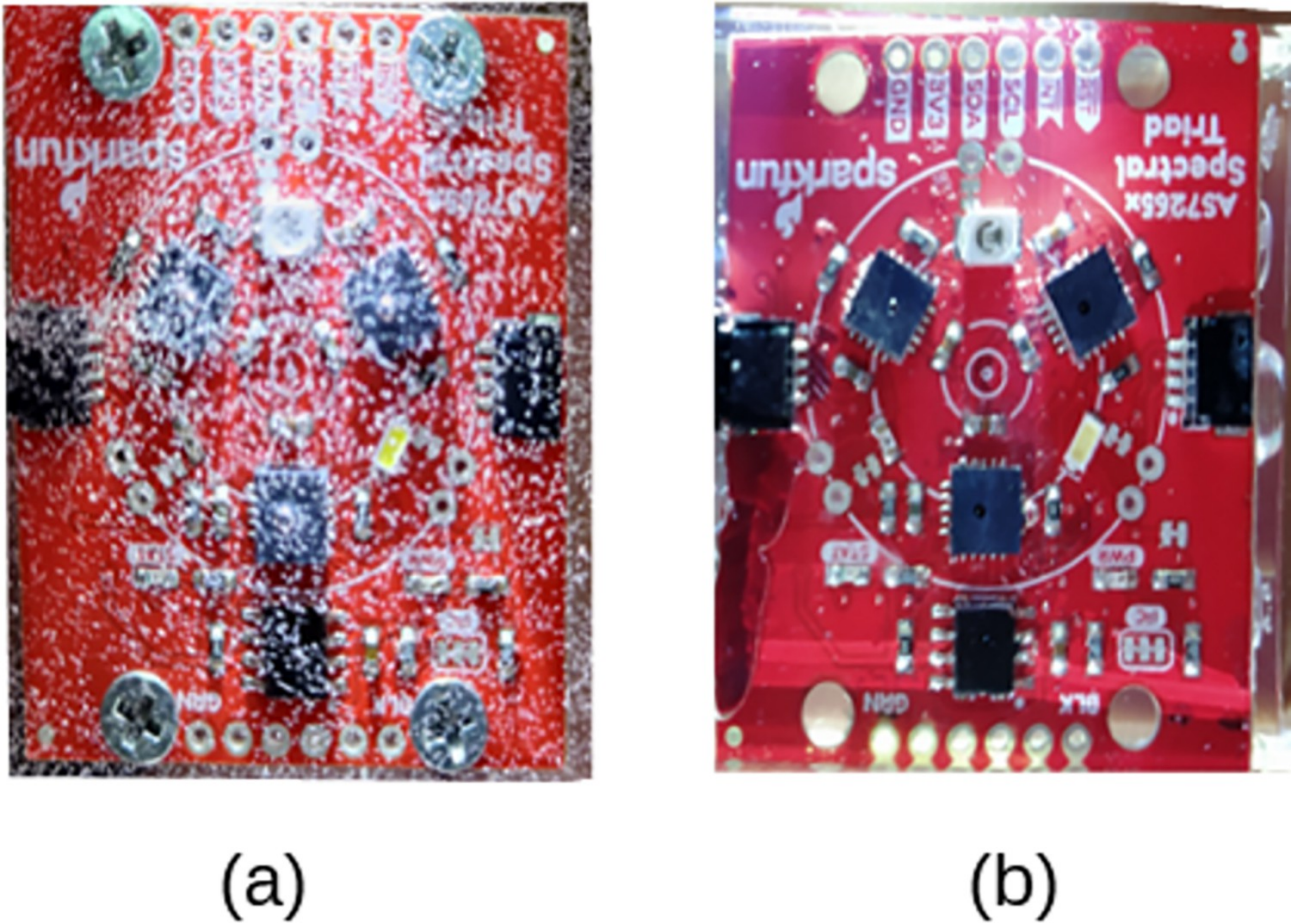
Resin selection tests for waterproofing the AS72365x. Image (a) shows the results of two-part epoxy resin and image (b) shows the one-part UV resin. An LED light is shined on the side of the casting to illuminate the air bubbles trapped in the casting.

**Fig 2:**
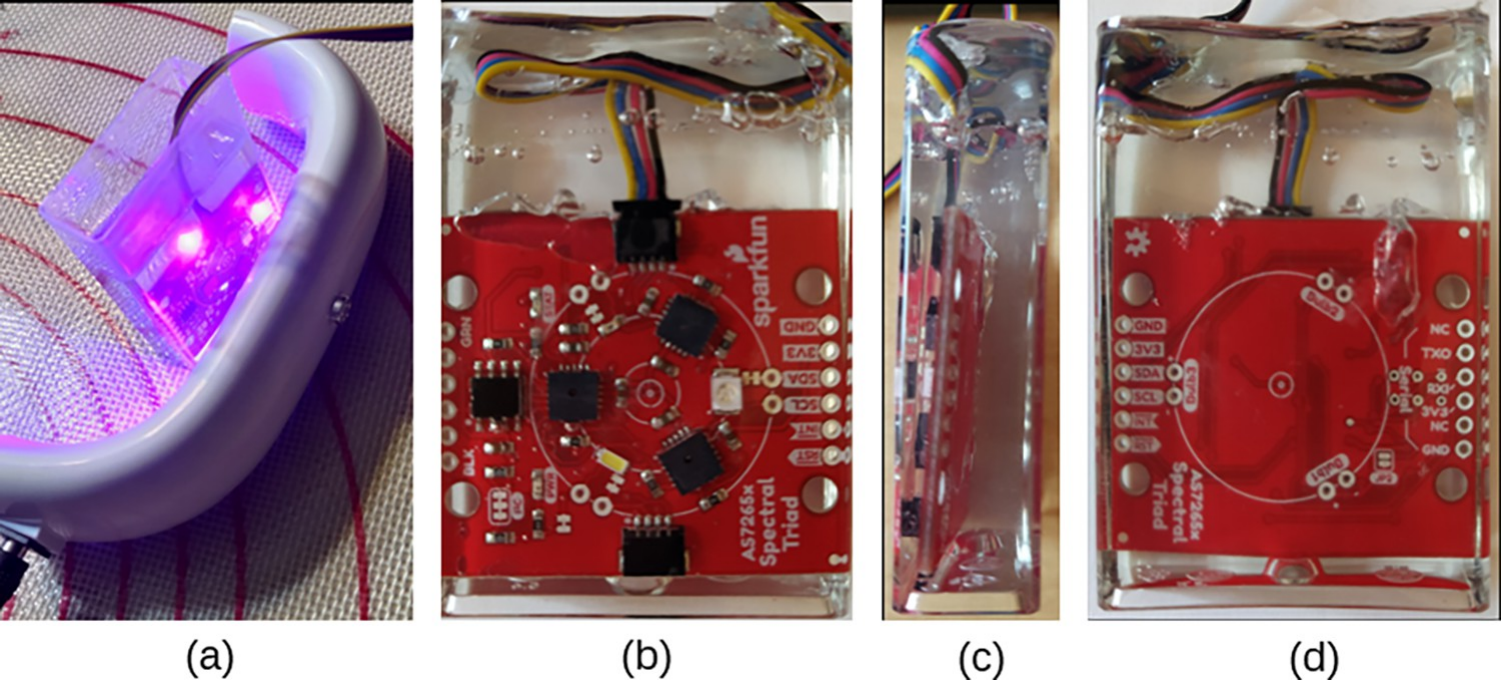
The final UV resin waterproofing cast of the AS7265x. The UV curing lamp used on the Tic Tac container (a). After curing the sensor was removed and is shown (b) front view, (c) is the side view, and (d) back view.

Resin casting was used to waterproof the sensors, addressing the issues of leakage caused using clear acrylic boxes identified by Stevens, Murray [10]. Each sensor was placed in a Tic Tac candy box, 37 x 14 x 62 mm dimensions filled with epoxy resin. A trial of two-part epoxy resin was first used but due to the large quantity of small air bubbles created in the mixing process, seen in Fig 1, it was abandoned, leaving four sensors. The remaining AS7265x sensors were cast in consumer grade UV resin from Blue Moon Studio, PA USA. A Tic Tac candy container, 37 x 14 x 62 mm, was used as a mold. The Blue Moon UV Resin is a one-part solution and almost no bubbles were present in the casting, seen in Fig 1. A Blue Moon Studio UV USB Curing Lamp was used to cure the resin. The lamp is 9 Watt and uses 395 nm spectrum. The UV light was applied to the mold five minutes on each side shown in Fig 2. After curing the casting was carefully extracted from the mold. Fig 2, shows the final product from front, side and back. The sensor uses the proprietary SparkFun Qwiic connector. Extra cable was laid laterally across the top and cast into resin to prevent moisture from wicking into the sensor, shown in Fig 2.

**Fig 3:**
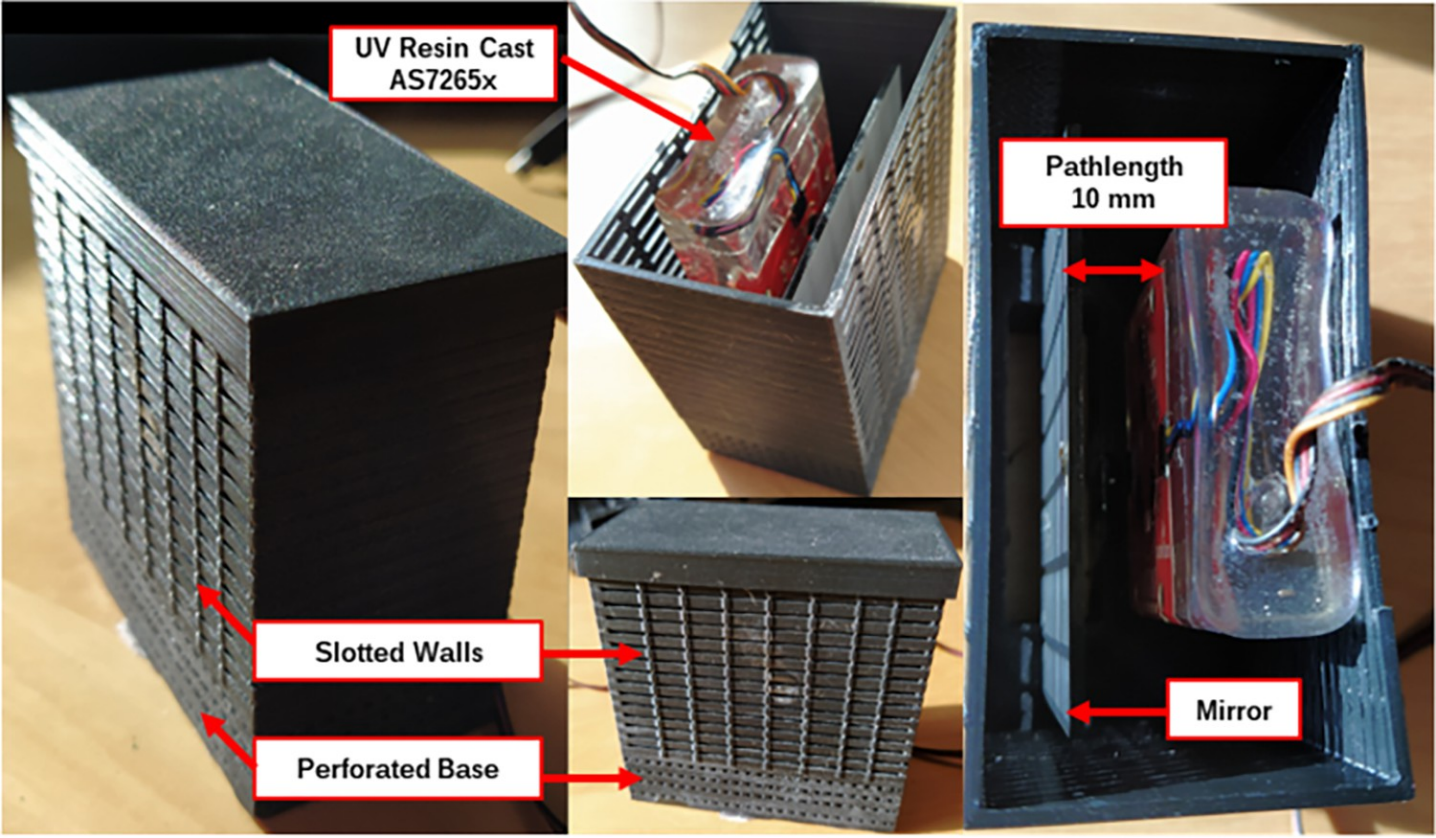
Transflective sensor housing with waterproofed AS7265x and aluminum mirror fixed at 10 mm distance from face of sensor.

Fig 3 shows the waterproofed AS7265x in a 3D printed housing with an aluminium mirror for transflective spectroscopy. Transflection gives the light two passes through the solution before being measured, making it ideal for measuring low concentrations, like in hydroponic nutrient solutions [39]. The 3D printed housing was printed with a consumer grade Creality Ender 3 FDM printer using PLA filament [40]. 10 mm was selected for the pathlength as this is the standard size of a cuvette used in lab spectrometers. As shown in Fig 3, the walls of the housing were slotted 1 mm apart and the base of the housing was perforated every 1 mm, this allowed for the nutrient to move freely through the housing while containing the onboard LED light. A lid was fixed to the top as well for the same purpose.

Consistency and functionality tests established the sensors ability to give consistent raw spectral intensity readings using the factory calibration to form a baseline. The raw spectral range measures the intensity of a particular wavelength between 0 and 65,535. An average home user will not have access to lab equipment for chemical analysis of the nutrient solution or sensor calibration, therefore the most important aspect is to establish sensor consistency rather than testing for sensor accuracy. Additionally, commodity sensors are precalibrated, and this testing regime aligns with the citizen science approach of the study.

All four sensors were fixed to the bottom of a 40 L black tank and raw spectral data was collected for three different conditions. First the sensors collected data from the dark empty tank with only ambient air. Next, 20 L of tap water was added to the tank. Finally, the tap water was replaced with 20 L of distilled water and the recording process was repeated. For each condition sensor measurements were recorded every six seconds for twenty minutes.

All sensors performed as expected, demonstrating the required functionality to provide consistent readings. As is seen in the Table 1, the SD and the SE are relatively low compared to the mean for all sensors in all conditions. Additionally, the difference between the minimum and maximum for all sensors was minimal. The sensors performance were not as consistent as the results shown for the same sensor quality tests of Stevens, Murray [10], this is likely attributed to the UV resin waterproof casting. First, the UV resin has a refractive index around 1.6 [41] compared to glass which has a refractive index around 1.5 [42]. Second, due to the casting process there were inconsistencies in the thickness of the UV resin across the face of the sensor. A standard and consistent casting process in the future would be recommended for future work. The minimal difference between the min and max and the relatively small SD support the selection of sensors and waterproofing method for the purpose of this study.

**Table 1:** Sensor consistency test comparing each sensor in three different conditions for 20 minutes.

		Mean	Std Dev	Std Err	Min	Max
S1	Empty	13019.59	22.67	2.03	12927.56	13056.77
	Tap	8373.503	305.80	27.35	8070.84	8855.34
	Distilled	13222.57	18.51	1.65	13150.22	13253.28
S2	Empty	36185.52	27.93	2.50	36037.69	36233.52
	Tap	27461.11	12.58	1.13	27434.60	27492.17
	Distilled	36185.52	27.93	2.50	36037.69	36233.52
S3	Empty	13059.09	23.80	2.12	12959.11	13131.57
	Tap	9222.813	3.20	0.29	9213.16	9229.93
	Distilled	13094.85	21.85	1.95	12925.50	13119.29
S4	Empty	20011.94	30.96	2.76	19807.44	20057.04
	Tap	20261.73	7.42	0.66	20244.22	20282.56
	Distilled	19674.01	19.13	1.71	19585.86	19724.85

### 2.2 Sensor system reliability evaluation

This section describes the reliability evaluation of the sensor system and the construction of the nutrient prediction models used for sensor calibration and deployment. The process is designed to thoroughly demonstrate the reliability of the sensor to predict the level in mL of hydroponic nutrient solution in a nutrient tank. Standard consumer grade hydroponic liquid nutrient solution typically comes in two parts to avoid nutrient precipitation, part A and part B. This study was carried out in Dubai, UAE in February of 2023 and uses the locally sourced Greenoponics Growth XL a nutrient solution from Greenoponics Agricultural Services, Dubai UAE. Growth XL Nutrient A contains Nitrogen, Calcium, and Iron while Growth XL Nutrient B contains Phosphorus, Potassium, Magnesium, Copper, Manganese, and Zinc.

Reliability evaluation consisted of three phases. All phases were carried out in the same nutrient tank used in the final experiment. The data points from each phase were collected every 10 seconds for six minutes. All phases used distilled water to eliminate any confounding factors of trace elements that may be contained in tap water. Upon adding each dose of nutrient, a circulation pump was activated for 30 seconds, after one minute EC readings were taken and the raw spectral values were recorded. Table 2 shows the testing methodology details for each phase of the test. Phase one and two examines the ability of the sensor to reliably predict the quantity of nutrient A and B individually and provides data to determine if there is an effect of the nutrient solutions on one another. Phases one and two use only 20 L of distilled water for testing due to limited supplies. Additionally, the 20 L volume is easily scalable and proportional to 40 L, making it a practical choice for testing purposes. Phase three used the same conditions deployed in the system to determine if the sensor system can detect the combined amount of mL added, as in final deployment of the system. The phases look at the effect of adding each nutrient separately and combined. The initial phase examines dosing nutrient A into the distilled water solution, followed by the introduction of nutrient B into the same solution. Phase two uses the same methodology but permuting the sequence of nutrients. Finally, phase three introduces both A and B simultaneously. In each phase of the study, nutrient concentration was quantified using two distinct measures: EC and the quantity of mL. The independent variables were comprised of the 18 spectral wavelengths detected by the AS7265x sensor.

**Table 2:** Description of the three phases of carried out for reliability testing of the sensor system.

Phase One	Into 20 L of distilled water, 25 mL of nutrient A is added in 1 mL doses. Next, 25 mL of nutrient B is added in 1 mL doses.
Phase Two	Into 20 L of distilled water, 25 mL of nutrient B is added in 1 mL doses, Next, 25 mL of nutrient A is added in 1 mL doses.
Phase Three	Into 40 L of distilled water, 2 mL of nutrient A and 2 mL of nutrient B are added separate but simultaneously until a total of 200 mL of nutrient has been added.

After reliability is established, the predictive model is constructed. At this point the entire MSH system is constructed, and tanks are put in place. 40 L of distilled water is added to the control and treatment tank. Next, 10 mL doses of nutrient, composed of five mL of nutrient A and five mL of nutrient B, are added to each tank until a total of 200 mL is added. After adding each dose of nutrient, the circulation pump is activated for 30 seconds and data is collected for six minutes in 10 second intervals. This data is used to produce the multiple linear regression (MLR) model for the final experiment. The coefficients and intercept are coded into the system and used to calculate the predicted quantity of nutrient levels. The next section presents the final experiment methodology used to test the MISH nutrient management system. A MLR was used to examine the AS7265x ability to predict EC and quantity of mL of phase one. Table 3 shows the MLR results for all the individual sensors for both the EC and the quantity of mL. The results showed that all sensors under all conditions produced significantly accurate models. A MLR analysis was performed on the combined dataset from all sensors, using EC as the dependent variable. The results produced a significantly accurate model F(14, 7065) = 3874.501, p < .001 with an R^2^ = .885.

**Table 3:** Phase one MLR results for reliablity testing using EC and mL.

**Phase 1 predicting EC**			
	**Nutrient A**	**Nutrient B added to A**	**Both A and B**
Sensor 1	F (18, 873) = 8964.56, p < .001 and R^2^ = .995	F (18, 873) = 8964.56, p < .001 and R^2^ = .995	f (18, 1752) = 2873.17, p < .001 and R^2^ = .967
Sensor 2	F (18, 868) = 13536.29, p < .001 and R^2^ = .996	F (18, 861) = 1032.74, p < .001 and R^2^ = .956	F (18, 1748) = 5669.80, p < .001 and R2 = .983
Sensor 3	F (18, 873) = 18756.07, p < .001 and R^2^ = .997	F (18, 860) = 359.97, p < .001 and R^2^ = .883	F (18, 1752) = 1936.05, p < .001 and R2 = .952
Sensor 4	F (18, 871) = 18113.40, p < .001 and R^2^ = .997	F (18, 862) = 872.85, p < .001 and R^2^ = .948	F (18, 1748) = 4165.39, p < .001 and R2 = .977
**Phase 1 predicting ML**			
	**Nutrient A**	**Nutrient B added to A**	**Both A and B**
Sensor 1	F (18, 873) = 12530.71, p < .001 and R^2^ = .996	F (18, 860) = 524.46, p < .001 and R^2^ = .917	F (18, 1752) = 3370.99, p < .001 and R^2^ = .972
Sensor 2	F (18, 868) = 19998.08, p < .001 and R^2^ = .998	F (18, 861) = 1145.17, p < .001 and R^2^ = .960	F (18, 1748) = 6849.74, p < .001 and R2 = .986
Sensor 3	F (18, 873) = 24547.12, p < .001 and R^2^ = .998	F (18, 860) = 364.38, p < .001 and R^2^ = .884	F (18, 1752) = 2283.38, p < .001 and R2 = .959
Sensor 4	F (18, 871) = 20276.67, p < .001 and R^2^ = .998	F (18, 862) = 866.73, p < .001 and R^2^ = .948	F (18, 1752) = 5021.68, p < .001 and R2 = .981

Subsequently, a MLR was conducted to determine if the sensors could reliably predict the mL of nutrient that was added. The results showed that with the combined dataset, the sensors were able to reliably create a significant F(18, 7065) = 4624.953, p < .001 model to predict the mL of nutrient added to the distilled water solution with an R^2^ = .902. Considering the EC and mL analysis, phase one results strongly support the sensor systems ability to reliably measure the change of nutrient concentration in distilled water. Next, phase two results are presented.

In phase two, Nutrient B was added to distilled water and before adding Nutrient A. Table 4 shows the MLR results for each sensor during each stage of the testing phase. The models are all statistically significant and strongly support the sensors’ reliability. EC was initially used to test if the four sensors could reliably predict the concentration of nutrient added to the distilled water. A MLR performed on the combined dataset of all four sensors using EC as the dependent variable and 18 spectral wavelengths as independent variables. The analysis showed the sensors are able to significantly F(14, 6755) = 4072.943, p < .001 predict the EC levels of nutrient added to distilled water, with R^2^ = .887.

**Table 4:** Phase two MLR results all with a p < .001 for the reliability testing using EC and mL.

Phase 2 predicting EC			
Sensor Number	Nutrient B	Nutrient A added to B	Both A and B
Sensor 1	F (18, 843) = 488.27, p < .001 and R^2^ = .914	F (18, 847) = 18841.90, p < .001 and R^2^ = .998	F (18, 1673) = 1414.54, p < .001 and R^2^ = .938
Sensor 2	F (18, 844) = 836.86, p < .001 and R^2^ = .945	F (18, 848) = 35446.61, p < .001 and R^2^ = .999	F (17, 1676) = 3261.04, p < .001 and R^2^ = .971
Sensor 3	F (18, 848) = 971.18, p < .001 and R^2^ = .955	F (18, 848) = 29684.95, p < .001 and R^2^ = .998	F (18, 1677) = 2248.50, p < .001 and R^2^ = .960
Sensor 4	F (18, 843) = 3086.74, p < .001 and R^2^ = .985	F (18, 842) = 64245.32, p < .001 and R^2^ = .999	F (18, 1668) = 5078.22, p < .001 and R^2^ = .982
			
Phase 2 predicting ML			
Sensor Number	Nutrient B	Nutrient A added to B	Both A and B
Sensor 1	F (18, 843) = 524.42, p < .001 and R^2^ = .920	F (18, 847) = 28500.35, p < .001 and R^2^ = .998	F (18, 1691) = 1842.11, p < .001 and R^2^ = .952
Sensor 2	F (18, 844) = 876.30, p < .001 and R^2^ = .947	F (18, 848) = 55216.14, p < .001 and R^2^ = .999	F (17, 1693) = 4059.59, p < .001 and R^2^ = .976
Sensor 3	F (18, 846) = 995.86, p < .001 and R^2^ = .956	F (18, 848) = 37724.98, p < .001 and R^2^ = .999	F (18, 1695) = 2821.59, p < .001 and R^2^ = .968
Sensor 4	F (18, 843) = 3303.82, p < .001 and R^2^ = .986	F (18, 842) = 79510.24, p < .001 and R^2^ = .999	F (18, 1686) = 6241.10, p < .001 and R^2^ = .985

The same analysis was conducted on the same data set to determine the ability of the 18 spectral wavelengths to reliably predict the amount of nutrient added to the distilled water. A MLR also produced a significant model F(13, 6755) = 5298.350, p < .001 and R^2^ = .911 that can reliably predict the amount of nutrient in mL that is added to the distilled water. Considering the results of the EC and mL analysis, phase two results strongly support the sensor systems’ ability to reliably measure the change of nutrient concentration in distilled water. Given the outcomes of the EC and mL analyses, the results from phase one provide robust evidence of the sensor system’s consistent capability to accurately gauge alterations in nutrient concentration within distilled water. Phase three results are presented next.

The models across phases one and two, presented in Tables 3 and 4 of the reliability evaluations, strongly support the use of the sensor for the purpose of nutrient management. Finally phase three examines the sensor reliability in an identical scenario to the final experiment.

Sensors 1 and 2 were placed in Tank 1 and sensors 3 and 4 in Tank 2. The rationale behind this decision resulted from the observation that Sensor 1 and Sensor 3 exhibited the highest levels of reliability, while Sensor 2 and Sensor 4 demonstrated the lowest. Therefore, it was decided to position one of the high-reliability sensors alongside one of the low-reliability sensors in each tank. The following test aimed to determine whether sensors mounted within a 40 L volume of distilled water, could accurately predict equal amounts of nutrient A and B added simultaneously.

A MLR was used to analyse the combined Sensor 1 and Sensor 2 data of Tank 1. The results produced a significant F (17, 3402) = 32176.99, p < .001 predictive model with a R^2^ = .994. Similarly, the data from both Sensors 3 and 4 in Tank 2 was analysed with a MLR and produced a significant F (13, 3412) = 43377.39, p < .001 predictive model with a R^2^ = .994. Table 5 shows the individual MLR results for each sensor. It is clear the sensors can monitor the change in nutrient concentrations in a range of situations.

**Table 5:** Phase three MLR models for each sensor.

Tank 1	Sensor 1	F (18, 1707) = 28271.48, p < .001 and R^2^ = .997
	Sensor 2	F (18, 1681) = 45334.18, p < .001 and R^2^ = .998
Tank 2	Sensor 3	F (18, 1708) = 29588.70, p < .001 and R^2^ = .997
	Sensor 4	F (18, 1674) = 19658.37, p < .001 and R^2^ = .995

Following the determination of the sensors’ consistent ability to generate predictive models in all three phases, a model for the final experiment was formulated. The results of the reliability evaluation strongly support the use of the AS7265x to use in the model building phase.

### 2.3 Model building

The system was fully prepared for the final experiment and both tanks filled with 40 L of distilled water, seen in Fig 8. The full details of the system build, including the hardware, software and architecture are in S1 File. Nutrients were added in 10 mL doses until 200 mL had been added to each tank. Each dose was composed of 5 mL of nutrient A and 5 mL of nutrient B, administered separately but simultaneously. Pumps were then activated for circulation and EC and spectral readings were taken.

First the data from Tank 1, containing Sensor 1 and Sensor 2 was analyzed. The combined sensor data produced a significant (F = (14,1438) = 19932.006, p = < .001) MLR model with an R^2^ of .995. However, there was a high collinearity between 535 nm, 560 nm, 610 nm, and 705 nm. Next the data from Tank 2, containing Sensor 3 and Sensor 4 was analyzed. The combined sensor data also produced a significant (F = (12,1439) = 25596.837, p = < .001) MLR model, with an R^2^ of .995. However, there was a high collinearity between 535 nm, 585 nm, 645 nm, 705 nm. 730 nm, and 760 nm. The collinearity was concerning, as 705 nm and 585 nm were among the most influential predictors in phase three of the reliability testing.

To address the issues with collinearity a forward stepwise MLR modeling process was used to select the most influential spectral wavelengths for model simplification. The sensor with the best fitting model and the fewest number of predictors was used for the final deployment in the system. Table 6 shows the final model results. Based on the reliability testing, all models were significantly accurate. In tank 1, Sensor 1 had the fewest predictors and an R^2^ of .997. Tank 2, sensor 4 had the fewest predictors and also an R^2^ of .997.

**Table 6:** Final model MLR results.

Tank 2	Sensor 1	F (11, 714) = 20963.393, p = < .001 and R^2^ = .997
	Sensor 2	F (13, 714) = 17264.355, p = < .001 and R^2^ = .997
Tank 1	Sensor 3	F (11, 714) = 18781.545, p = < .001 and R^2^ = .997
	Sensor 4	F (9, 717) = 26560.535, p = < .001 and R^2^ = .997

Based on the results in Table 6 Sensor 1 was selected for Tank 1 having 11 predictors and Sensor 4 was selected for Tank 2, having 9 predictors. Further supporting this selection, both models included 705 nm and 585 nm, consistent the reliability tests. Table 7 shows the final models that were used for the sensor and the tanks they were assigned to.

**Table 7:** MLR models built and used in the final experiment. The control tank used sensor 4 and the treatment tank used sensor 1. For accuracy the model the coefficients must be coded using all six decimal places.

Control—Tank 1			Treatment–Tank 2		
Sensor 4			Sensor 1		
	Coefficient	Sig		Coefficient	Sig
(Constant)	907.663994	< .001	(Constant)	963.043834	< .001
585 nm	-6.173363	< .001	705 nm	2.345823	< .001
560 nm	2.962786	< .001	730 nm	2.150208	< .001
705 nm	2.233135	< .001	760 nm	-1.316648	< .001
810 nm	0.380447	< .001	585 nm	-1.219572	< .001
610 nm	-0.115807	0.002	645 nm	0.741491	< .001
900 nm	-0.112201	< .001	610 nm	-0.164956	< .001
535 nm	-0.018315	< .001	535 nm	-0.16176	< .001
410 nm	-0.017414	< .001	510 nm	0.133431	< .001
510 nm	0.017289	0.001	435 nm	-0.041807	< .001
			410 nm	-0.006562	< .001
			460 nm	0.005862	0.004

### 2.4 Final experiment methodology

The final experiment focuses on evaluating the MISH system ability to manage the hydroponic nutrient solution using the AS7265x and the trained prediction model to recommend the quantity of nutrient to be added. The purpose of the study is to determine if the proposed system can manage the cumulative nutrient level. Therefore, only the management of the combined nutrient levels of A and B are recommended, monitoring and managing individual nutrients is beyond the scope of this study. The experiment is conducted using a 30-day grow of lettuce in a real-world scenario in a residential apartment in Dubai UAE during the months of February and March 2023. A control tank with no nutrient management, in which nutrients were added once but not maintained, is compared with a treatment tank that uses the nutrient management system.

**Fig 4:**
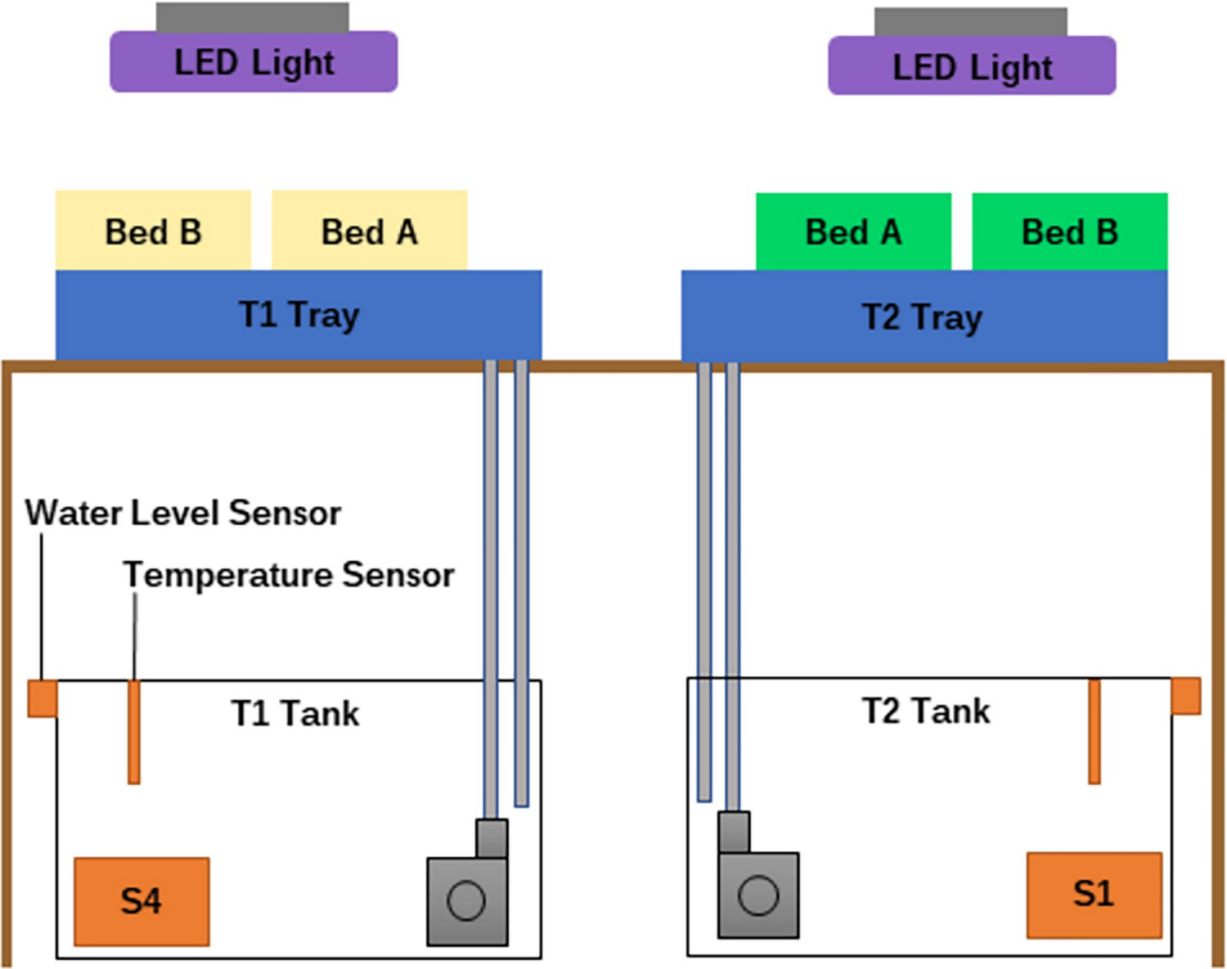
Final experiment T1 is the control and T2 is the treatment. S1 and S4 are calibrated sensors at the bottom of the tank.

The experiment set up shown in Fig 4, uses a set of identical of 40 L black, light blocking tanks, with secure lids, to prevent the algae growth and prevent interference with the AS7265x are used for control and treatment. The AS7265x sensors are mounted to the bottom of the tank near the pump, as shown in Fig 4. A water level sensor is fixed to the outside of the tank at the level of 40 L. Distilled water is added at the end of every day to provide a stable consistent environment for sensor reading as identified in [10]. The full MSHS system details, including the software and hardware architecture as well as the recommendation algorithm can be found in S1 File. Daily manual readings for EC are recorded and manually input into the system. The pH and the temperature are monitored and recorded. The thermostat in the apartment was set to 25°C.

A flood tray is attached to each nutrient tank at top the flood table. Two beds measuring 70 cm × 19 cm filled with perlite and topped with coco coir are placed in each flood tray. A 25-watt Dynamax pump was used in each tank to flood the beds for 20 min every 12 h. The beds are swapped on day 15. Two full-spectrum LED grow lights, measuring 300 mm × 240 mm, are fixed above the trays. The height and intensity of the light was adjusted to meet the recommended amount of photosynthetic active radiation of 14 daily light integral [43,44] using an Apogee SQ-520 quantum sensor.

All equipment was rinsed with distilled water before starting the experiment. The beds and media were rinsed with boiling distilled water and left to rest 24 h before starting the system. The nutrient tanks were filled with distilled water and the pump cycle ran for 24 h. Each tank was then topped up with distilled water. Next the nutrient A and B was added to tank in equal proportion until EC reached 1000 EC. Finally, six, Ten-day-old lettuce seedlings (Lollo bionda), were weighed and planted in each bed. On day 15 beds A and B were swapped for each bed. At the end of 30 days the plants were harvested, weighed, then dried in an oven at 80° C for 72 h and weighed again.

## 3. Results

The final experiment was conducted in a real-world application. Tank 1 served as control and Tank 2 was the treatment. Sensor 1 was mounted in treatment tank and Sensor 4 was mounted in control tank. Both sensors were connected to the MISH system. The corresponding prediction models were coded into the MISH system software to predict the mL quantity of combined nutrient in each tank. The predicted mL readings from Sensor 1 were used to adjust nutrients for the treatment tank across the 30-day experiment. Sensor 4 was used to predict the mL readings for data collection purposes.

To begin the final experiment each tank was set up and filled with 40 L of distilled water. Next, 100 mL of nutrient A and 100 mL of nutrient B were added to the tanks simultaneously, equalling an EC of 1000 μS/cm. The daily EC reading were recorded along with the mean predicted mL for each tank. Distilled water was added to both tanks every day to maintain 40 L.

**Fig 5:**
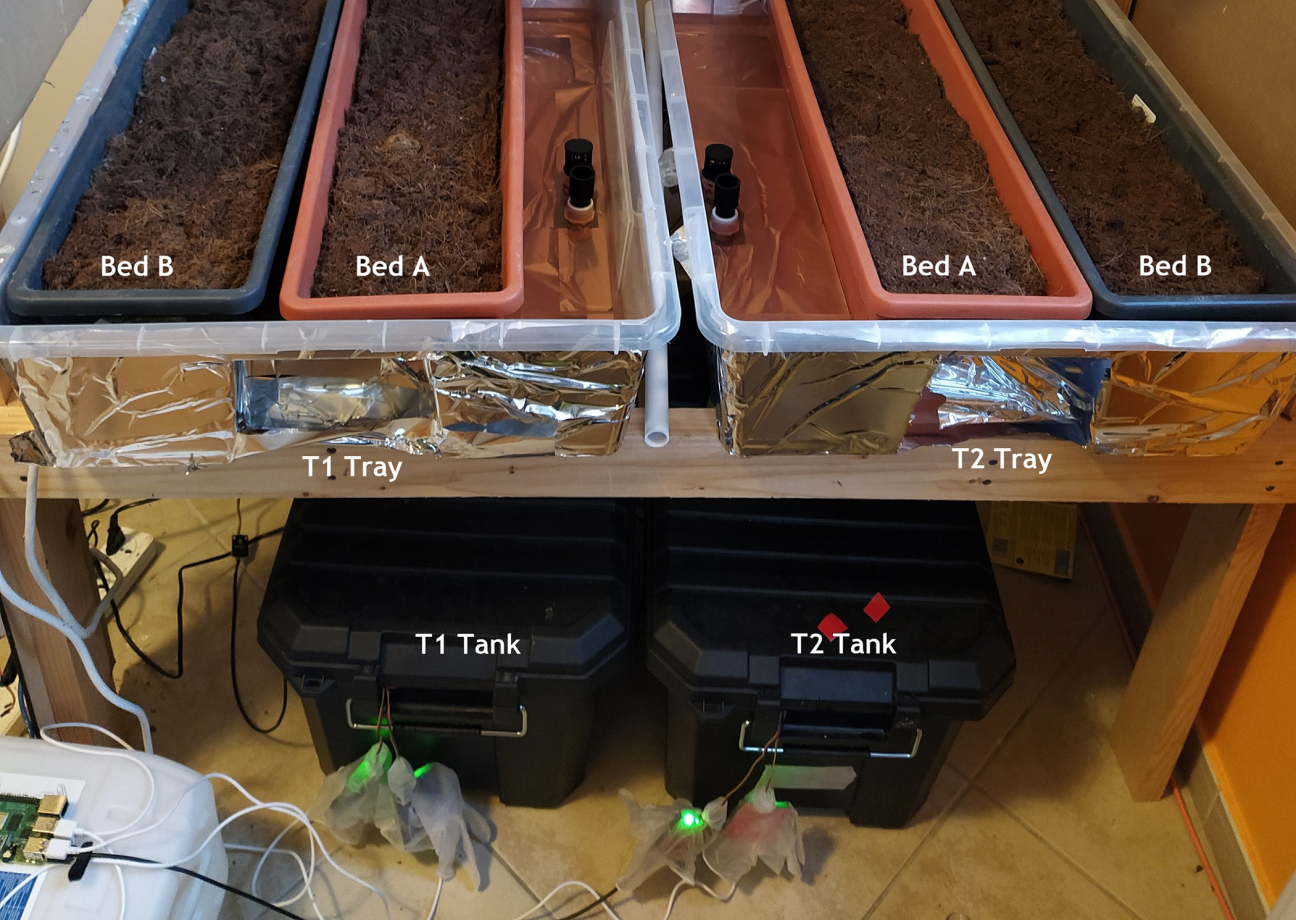
The final experiment setup. Depicted is the initial flooding just before the baseline data was collected. A cardboard barrier was affixed to the front and sides, to eliminate light leakage.

To adjust the nutrients for the treatment tank, the predicted mL had to be subtracted from the baseline. Prior to starting the actual grow for the final experiment the baseline had to be established. After the nutrients were added to each tank, the beds were flooded for 20 minutes then drained. Fig 5, shows the experiment set up as the baseline data is being collected. The predicted mL was recorded every 10 seconds for both sensors for 24 hours. Table 8 shows the descriptive statistical analysis used to derive the baseline. Based on the analysis the baseline for the treatment is 183 mL and the baseline for the control was 172 mL with only 11 mL difference.

**Table 8:** Baseline means of 24 hours of predicted readings after setting up the tanks.

	Baseline Mean of Predicted mL	Standard Deviation	Max	Min
Treatment	182.94	2.19	218	169
Control	172.20	4.43	189	160

**Fig 6:**
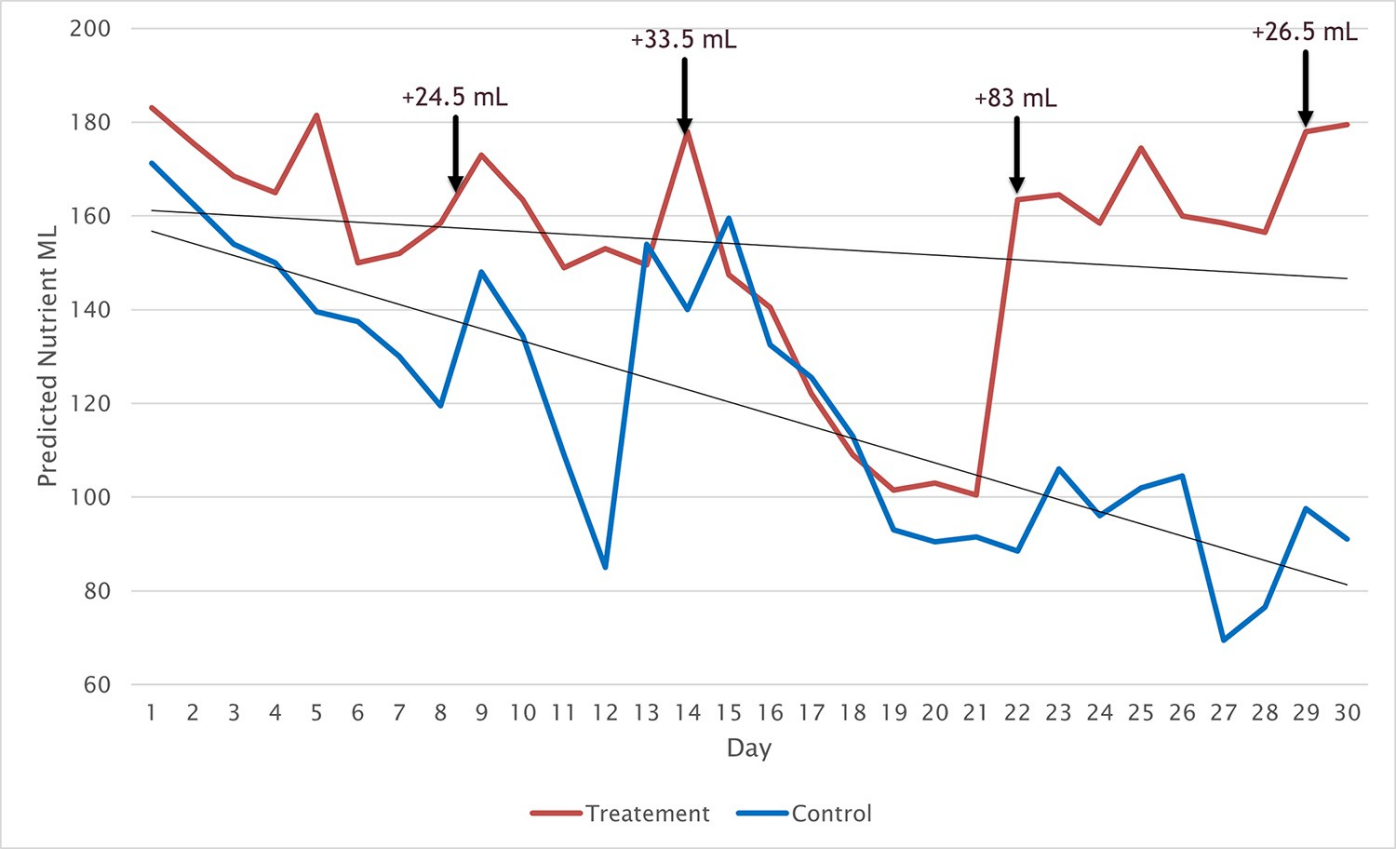
Predicted mL for treatment and control over 30 days with trendlines. Nutrient adjustments from Table 9 are indicated on the graph.

Fig 6 shows the daily mean for the predicted mL for both the control and treatment plotted over 30 days. The overall trendlines shown in Fig 6 demonstrates that the treatment has a more gradual decline than the control. An independent t test was conducted to compare the means between the Control (M = 156.46, SD = 42.40) and the Treatment (M = 117.68, SD = 28.89) across the 30-day experiment. The analysis shows a significant t(464213) = 364.004, p = < .001) difference between the two.

**Fig 7:**
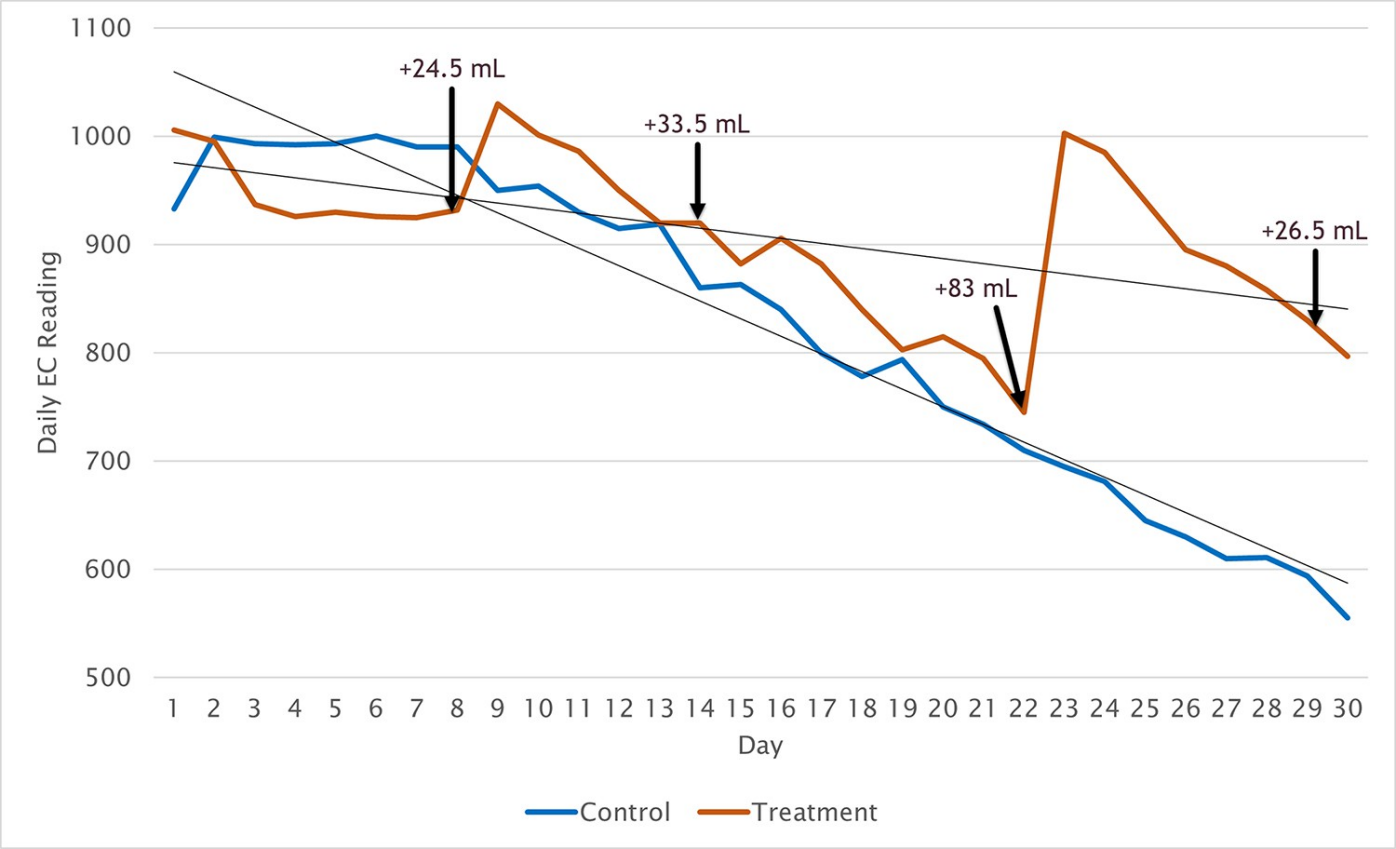
Daily EC readings for both tanks across the 30-day experiment with trendlines. Nutrient adjustments from Table 9 are indicated on the graph.

Additionally, an analysis was performed on the change of the EC over the course of the experiment. Fig 7 shows the daily recorded EC, plotted over 30 days, along with a trendline overlay. Both treatment and control have an overall downward trend, however the decline for the control is more prominent. The EC for the treatment has more pronounced fluctuations than the control, due to the nutrient adjustments. To verify the differences a comparison between the 30 day means of the EC was conducted. The treatment (M = 899.815, SD = 74.71) and the control (M = 823.70, SD = 145.74) showed a significant (t(464213) = 223.92, p = < .001) difference. This is as expected and consistent with the data in Fig 6, supporting the system’s ability to track and measure the changes of nutrient in mL.

Finally, an analysis of the control tank’s daily EC and the daily predicted mL mean using Pearson’s correlation shows a significant positive relationship r(30) = 0.77, p < 0.001. An analysis of the treatment tank daily EC and daily predicted mL mean also significantly indicates a positive relationship r(30) = 0.53, p = 0.002. This indicates that the predicted mL model tracks the changes in EC, affirming the model’s predictive ability.

Using the MSH nutrient recommendation system, four nutrient adjustments were made to the treatment tank over the course of the 30-day experiment. Table 9 shows the day and the amount of nutrient added. These additions can clearly be seen in both Figs 6 and 7. After the adjustment was made the EC and predicted mL both increase. Nutrient was dosed in equal parts Nutrient A and Nutrient B for each adjustment. A total of 167.5 mL of nutrient was added.

**Table 9:** Timing and volume of hydroponic nutrient added to the treatment tank based on the MISH system recommendation.

Day in the experiment	Amount of Nutrient in mL
8	24.5
14	33.5
22	83.0
29	26.5

To address any potential confounding factors, temperature and pH were both analysed. The temperatures for the two tanks were compared to eliminate confounding factors. Tank 1 (M = 24.48, SD = .58) had a significant (t(92773) = -25.10, p < .001) marginally lower temperature than Tank 2 (M = 24.57, SD = .61) of .09° C, demonstrating a stable comparable temperature. The pH for the two tanks were also compared to discount confounding factors. Tank 1 (M = 6.16, SD = .32) had a significant (t(58) = 2.57 p = .006) marginally higher mean pH than Tank 2 (M = 5.97, SD = .24).

**Fig 8:**
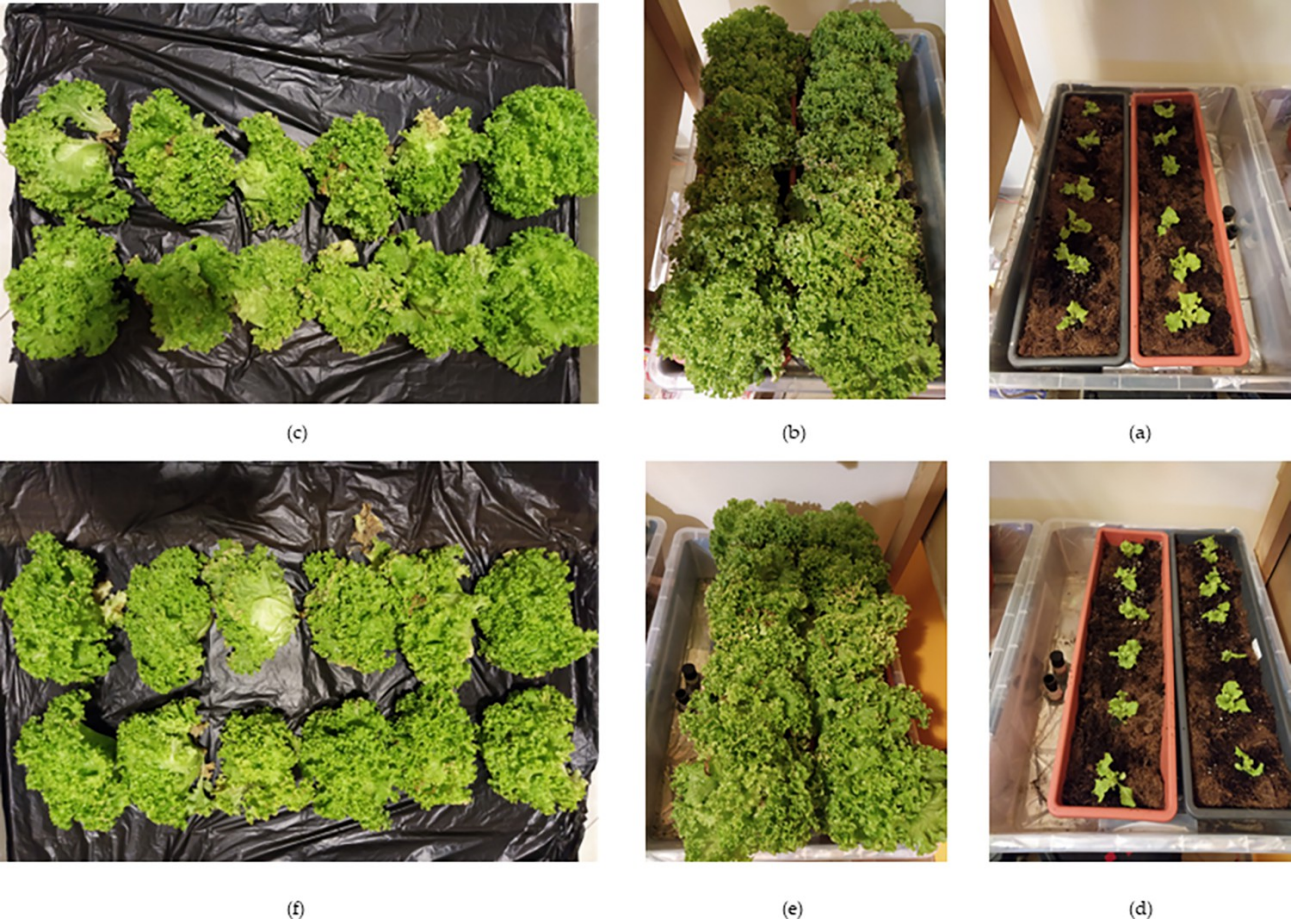
Growing phase of the final experiment: The control plants after transplant **(a)**; the control plants the last week **(b)**; the control plants harvested ready for weighing **(c)**; the treatments plants after transplant **(d)**; the treatment plants in the last week **(e)**; the harvested treatment plants ready for weighing **(f)**.

The grow results, in Fig 8 shows the plants for both the control and the treatment during the first week, last week and at harvest. At week one the seedlings appear similar for both groups. The growth for both are similar with only slight visible differences in the middle of the control tray as seen in Fig 8B.

Seedlings for both control and treatment were weighed before transplanting. An independent samples t-test was conducted to see if there were significant differences of the means of the control (M = 2.14,.SD = 0.79) and treatment (M = 2.04,.SD = 0.43, n = 12). The test t(22) = 0.191, p = 0.85, indicated no significant difference between the seedling weights upon transplanting.

After 30 days the plants were harvested and weighed again to examine the effects of the treatment. First the fresh weight was examined. An independent samples t-test was used to determine if there was a significant mean weight difference between the control (M = 83.30,.SD = 16.46) and treatments (M = 126.1333, SD = 18.10). The t-test t(22) = -6.06, p < .001, indicates there is significant difference in the mean fresh weights of the control and treatment.

After this the plants were dried in an oven for 72 hours and weighed again. It is important to note that the study lacks pre-growth dry weights for comparison and no replication in the dry weights. An independent samples t-test was also used to determine if there was a significant difference in the mean dry weights of the control (M = 4.99,.SD = 0.14) and treatments (M = 6.29, SD = 0.37). The t-test t(22) = -11.61, p < .001, indicates there is also a significant difference in the mean dry weights of the control and treatment. While the dry weight finding is not generalisable it does provide insight and lend support to the validity of observed differences between the treatment and control groups. Table 10 shows the total weights, fresh and dry, for both groups. It is clear from the statistical analysis and the sum weights that the treatment produced more biomass than the control. This finding is expected and in line with fact there was more nutrient available for the plants to use, leading to increased weight in the treatment.

**Table 10:** The total weight for the 30-day experiment.

		Fresh Weight	Dry Weight
Control	Total Weight	83.3 g	5 g
Treatment	Total Weight	126.1 g	6.3 g

In summary, the results show that the nutrient management system effectively managed the nutrients need to sustain growth in a microscale hydroponic system. These findings are interpreted in the next section.

## 4. Discussion

This study presents a complete system architecture, model construction, and sensor calibration methodology, aligned with a citizen science approach, that a home user can implement to manage nutrients without using EC or ISEs. The results from the final experiment and sensor model building overwhelmingly show that an inexpensive commodity IoT spectral sensor can provide a sufficient, robust alternative method to EC and ISEs, adding to the findings of Stevens, Murray [10] and Silva, Löfkvist [35].

This study also presents a novel, effective, and reliable way to measure nutrients for easy application by measuring hydroponic nutrients in mL instead of EC readings or parts per million. All MLR models for the sensor quality testing and final deployment were significant. Among all the models produced, sensor three in reliability testing phase one performed the worst when adding nutrient A to B, with an R^2^ = 0.883. The performance was similar for the same sensor when adding B to A, with an R^2^ = 0.884, suggesting an issue with the sensor. The final deployment did not use sensor three because thorough sensor testing identified its inferiority, evidence of the rigorous methodology. It also highlights an issue. Models were more accurate when produced from adding just one nutrient solution or both simultaneously, suggesting that EC is not the most reliable dependent variable. There is also the case that because forward stepwise regression was used, the models were overfitting and looked usable, but when applied, they fell short in accuracy. The predicted mL fluctuated widely for treatment and control in the final experiment but was reliable enough to track changes in the nutrient content effectively.

The system successfully tracked the mL quantity of nutrients in both tanks over the 30-day experiment, which is clearly demonstrated in the strong correlations of the predicted levels of mL to the EC readings. For the control tank, there was a strong positive significant correlation between EC and predicted mL r(30) = 0.77, p < 0.001. The findings were similar for the treatment r(30) = 0.53, p = 0.002. While the positive correlation for the treatment is not as strong as the control, the result of the adjustments and the method of recording the EC likely played a role.

The adjustments are far more pronounced in the predicted mL than in the EC. However, the EC treatment data reflects the predicted mL on day 22, clearly seen by comparing Figs 6 and 7. While visually minor similarities exist between the changes in the predicted mL in the control and treatment tank a, Pearson’s correlation r(231982) = .119, p < .001 between the two reveals no substantial relationship. This correlation was expected, considering the similar fluctuations earlier in the experiment and the vast differences after the adjustments to the treatment were made. Additionally, the wide fluctuations in the control and treatment tanks for the predicted mL align with the findings of Stevens, Murray [10] that also used a similar application of a spectroscopy sensor. However, they reported lower fluctuations, which is expected as the present system examines the sum of all nutrients in the tank instead of only N. Since the nutrient is at rest most of the time, it raises the question of whether there is a need to continually monitor nutrients in the nutrient tank of an ebb and flow system.

The analysis of the experiment plant growth further shows the effectiveness of the nutrient management system. The average fresh weights for the seedlings showed no significant difference upon transplanting. After the grow, the control plants yielded significantly less fresh weight and dry weight than the control, the expected result. The tank with more available nutrients will produce more biomass [10].

The total recommended nutrient added was 167.5 mL, comprised of 83.75 mL each of nutrients A and B. The baseline was 183 mL for the treatment tank, and the mean last reading for the treatment was 182, a remarkable finding and provides strong evidence of the system’s successful performance. The baseline for the control was 172 mL, and the mean last reading was 91 mL. The tests of temperature and pH between treatment and control tanks discount confounding factors that would explain the difference in the nutrient uptake of the plants, which leaves only the system-recommended adjustments accounting for the increased weight in the treatment plants.

Comparable studies were challenging to find, given the nature of the experiment testing the novel nutrient management system. Of the previous studies examined, only one study reported an analysis of plant growth [32] that would be semi-comparable. Jung, Kim [32], lettuce yield for 56 heads of lettuce was an average of 52.6 g per head, using their ISE based nutrient management system. The average for the 12 heads of lettuce produced in this study was 10.5 g per head. There are number of reasons for the difference. Firstly, there was a difference in the sample size, the amount of lettuce Jung, Kim [32] grown was greater. Secondly, the strain of lettuce was different, which can influence growth rate. Lastly, the hydroponic system used in Jung, Kim [32] was markedly different, considering the variety of variables such as temperature, light intensity, and nutrient composition. These factors can greatly impact growth and yield. Taking into account these factors, it would be inappropriate to directly compare the average yields. Given the limited availability similar studies, the most suitable comparison is to analyse the sensor accuracy.

The most similar studies are Silva, Löfkvist [35] and Silva, Queirós [45]. These studies used study a patented mobile mini spectrometer based on STS-UV-L-50-400 Ocean Insight spectrometers. Silva, Löfkvist [35], using an undisclosed self-learning AI model, achieved predicted: N correlation of 0.99, P correlation 0.92, and a K correlation of 0.98. This is high, compared to the results of this study, a correlation of 0.77. While Silva, Löfkvist [35] examined individual nutrients, and this study examined the overall nutrient content the comparison is promising. The models applied for this study achieved a 0.77 correlation, close to what was achieved, in lab conditions [35]. The sensor used in this study is constructed with an open source commodity grade IoT sensor using basic MLR and still achieved a comparable level of accuracy to Silva, Löfkvist [35].

The results are even more noteworthy comparing the initial models to Silva, Queirós [45] which used the same AI model and a further developed mini spectrometer. Their initial models were trained on a custom created nutrient solution and reported N: R^2^ = .98, P: R^2^ = 0.84, K: R^2^ = 0.99 [45]. When that model was applied to real world samples from various commercial greenhouses only the N retained the high prediction levels, N: R^2^ = 0.94, P: R^2^ = 0.68, K: R^2^ = 0.67 [45]. Compared to the initial models produced in this study using a simple, less costly solution of R^2^ = 0.99. Considering the strong initial models presented in this study, built with store bought fertilizer and the applied performance of the nutrient management system highlight the potential of the simple, inexpensive system for managing hydroponic nutrient.

Further, this study contributes to the work of Stevens, Murray [7] and Stevens, Murray [10] by establishing a successful MSH technique using a simple flood and drain system with a black tank to prevent algae and flooding the lettuce every 12 hours for 20 minutes. This technique will produce a harvest of lettuce in 28 days. Additionally, this research supports the sensor design, which was thoroughly tested and proven to work for measuring and tracking nutrient content in a hydroponic solution. The sensor housing design provides simple transflective spectroscopy for cheap home use, thus eliminating the need for complicated system apparatus [e.g. 31,32,35]. Furthermore, a cheap, durable waterproofing method using everyday items such as a candy box for a mould and resin from a hobby store can produce a reliable sensor that does not compromise accuracy.

Several limitations need to be considered for future work. Firstly, as in Jung, Kim [32], there were issues within the software system. The date of the adjustment needed to be coded correctly. Instead, the date the recommendation was given was used to determine when to give the following recommended adjustment. The first recommendation was given on the seventh day but was made on the morning of the eighth day. This issue can be addressed through automation but highlights the robustness and durability of the system in a real-world application where users make mistakes. Also, lettuce is a very robust plant, and there is a case for not adjusting anything considering the results of the control tank. As long as there is sufficient nutrient at the beginning, letting the plants grow without dosing them is viable. This outcome aligns with the findings of Stevens, Murray [10]. An alternative would be examining how this system can be used to enhance the reuse of hydroponic solution.

The lack of uniformity in sensor waterproofing potentially caused the sensors to have very different models in the same tank. The use of factory calibration may have also caused this. Also, the natural decrease in the nutrient only started in the second half of the final experiment. A longer duration with a lower starting EC would have given more insight into the management system’s capabilities. Future studies must establish a repeatable waterproofing method and customize the calibration before starting.

The scope for future work would be three-fold. First, models produced with artificial intelligence and machine learning can be used to distinguish between nutrient A and nutrient B, as demonstrated in Silva, Queirós [45]. Advanced models would enable the precise dose of the nutrients as needed. The next would be to automate the system with peristaltic pumps. This automation would simplify the model-building process.

## 5. Conclusion

In conclusion, the application of IoT spectroscopy has great potential for simplifying nutrient management for home users of MSHS. Previous work in this area has used expensive sensor systems that employ ISE probes or proprietary spectroscopic sensors. Both methods are unsuitable for Microscale Smart Hydroponic Systems. A waterproofed inexpensive IoT spectroscopy sensor was developed, and a three-phase model building process was carried out. The models were constructed to predict the mL of store-bought hydroponic nutrient in the solution with an R^2^ = 0.997. The model was then deployed in a real-world experiment comparing the performance of the nutrient management system with a control group. The results showed a significant t(22) = -6.06, p < .00 difference between the plant growth and the control (M = 83.30,.SD = 16.46) and treatments (M = 126.1333, SD = 18.10). There was also a strong correlation between the EC and the predicted mL of 0.77. Through the use of low-cost IoT grade uncalibrated sensors, when augmented by the implementation of a software model, has the ability to bring commercial-scale nutrient management into the domain of microscale hydroponic systems."

## Supporting information

S1 File Supplemental system construction details. https://doi.org/10.6084/m9.figshare.25434514.v2.
(DOCX)

S1 Table Sensor consistency testing complete dataset. https://doi.org/10.6084/m9.figshare.24772905.v4.
(XLSX)

S2 Table Sensor reliability testing phases complete dataset. https://doi.org/10.6084/m9.figshare.24782601.v3.
(XLSX)

S3 Table Predictive mL modelling complete dataset. https://doi.org/10.6084/m9.figshare.24784434.v3.
(CSV)

S4 Table Baseline establishment complete dataset. https://doi.org/10.6084/m9.figshare.24784440.v3.
(CSV)

S5 Table Plant weights complete dataset. https://doi.org/10.6084/m9.figshare.24784266.v3.
(XLSX)

S6 Table Final 30-day experiment complete dataset. https://doi.org/10.6084/m9.figshare.24784497.v3.
(ZIP)
