# Supplementary material for: A low-cost spectroscopic nutrient management system for Microscale Smart Hydroponic system
Source: PLoS One. 2024 May 8;19(5):e0302638. doi: 10.1371/journal.pone.0302638 (PMC11078404; doi:10.1371/journal.pone.0302638)

**Supplemental Information**

**S2 File**

*Details of the hardware and software system construction used in the nutrient management and MISH system*

Stevens et al.

The MISH system that was constructed was a complex IoT system with multiple sensing, actuating ans service components. The following section explains the system components and presents the hardware and software architecture. To align with the principles of citizen science, the study used hardware components comprised of commodity consumer grade IoT equipment and all software was free open source.

Figure 1 shows the IoT MISH system hardware design. Raspberry Pi 4B(RPi) serves as the gateway and host for the required services. Each SparkFun Triad Spectroscopy Sensor-AS7265x connects to an Arduino Uno form factor, SparkFun RedBoard Qwiic board. The AS7265x connects using an I^2^C bus via the SparkFun’s proprietary Qwiic adapter. Each AS7265x has the same I^2^C address and cannot be connected to the same Arduino directly. Each sensor has its own Arduino in place of an I^2^C multiplexer. Due to limitations in budget and local availability a decision was made to use individual Arduinos over the multiplexer. Each Arduino connects via USB to the RPi gateway that exchanges data with the another RPi on Wi-Fi 2.4 ghz. For monitoring temperature and water level, shown in Figure 1, a generic DS1820 temperature sensor and a DF Robot Non-contact Liquid Level Sensor XKC-Y25-T12V connects to an Arduino brand Arduino Uno’s using digital general purpose input output (GPIO) pins. A non-contact liquid level sensor is selected to avoid interference that in tank sensors are susceptible to as identified in Stevens et al. (2023). These Arduinos then connect to the RPi using USB. Finally, the GPIO pins from the RPi connected to three Fotek SSR 40 DA relays. The relays were used to actuate the water pumps and the LED lights. The tanks share the same light source so only one relay was needed for the lights.

Figure 1-The hardware architecture for the system showing the infrastructure of the IoT components.


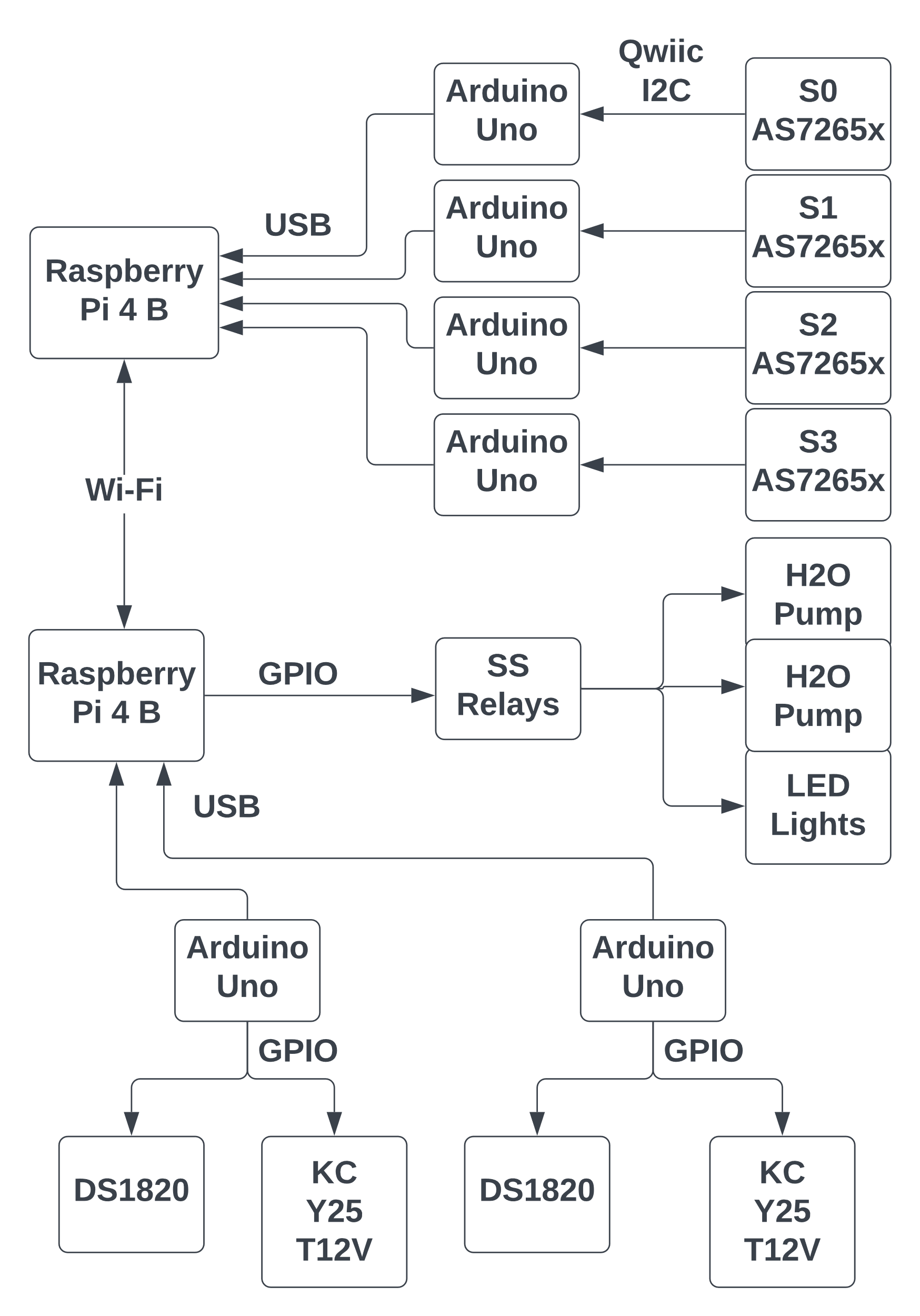


Figure 2 shows the services that were installed on each RPi and how they interact. The operating system installed on the RPi is Raspbian, a Debian Buster 10 based linux distrubtion customized for the RPi. The main application is built on Node-Red 3.0.2 that uses NodeJs version 16.16.0. Node-Red is a visual programming tool for event driven applications. It has access to wide variety of ready built packages, called nodes, that allow for easy interaction with other services and allows direct access to RPi GPIO pins. Node-Red is used to connect and control all of the sensing and actuation. In addition to an extensive library of existing packages, Node-Red also has an interface for direct JavaScript code for building complex applications. Data is exchanged between the two Node-Red instances using Mosquitto 2.0.11, a Message Queuing Telemetry Transport (MQTT) broker. The MQTT broker allows for both sending and receiving data using a simple subscribe and publish architecture. Once the data has been processed in Node-Red it is written to the respective database.

Two separate databases are used in the application, a structured query language (SQL) database, MariaDB 10.6.12, and a time series database, InfluxDB 1.8.5. The SQL database serves as the central repository where all the data is aggregated and stored together for easy analysis later. The timeseries database is used to store the data related to nutrient level predictions. InfluxDB does not require tables and schemas to be created in advance, data can be written with a simple key value pair. This feature is ideal for storing nutrient level predictions. The easy storage feature saves time in creating and testing multiple models for nutrient prediction. The databases are installed on separate RPi’s to accommodate for the growing database size in relation to the storage size limitations of. Both databases easily integrate with Grafana 8.1.5 dashboard. Grafana dashboard connects to both databases directly and uses the tables to create a user dashboard for easy monitoring.

Figure 2. Software architecture for the services used on each Raspberry Pi 4.


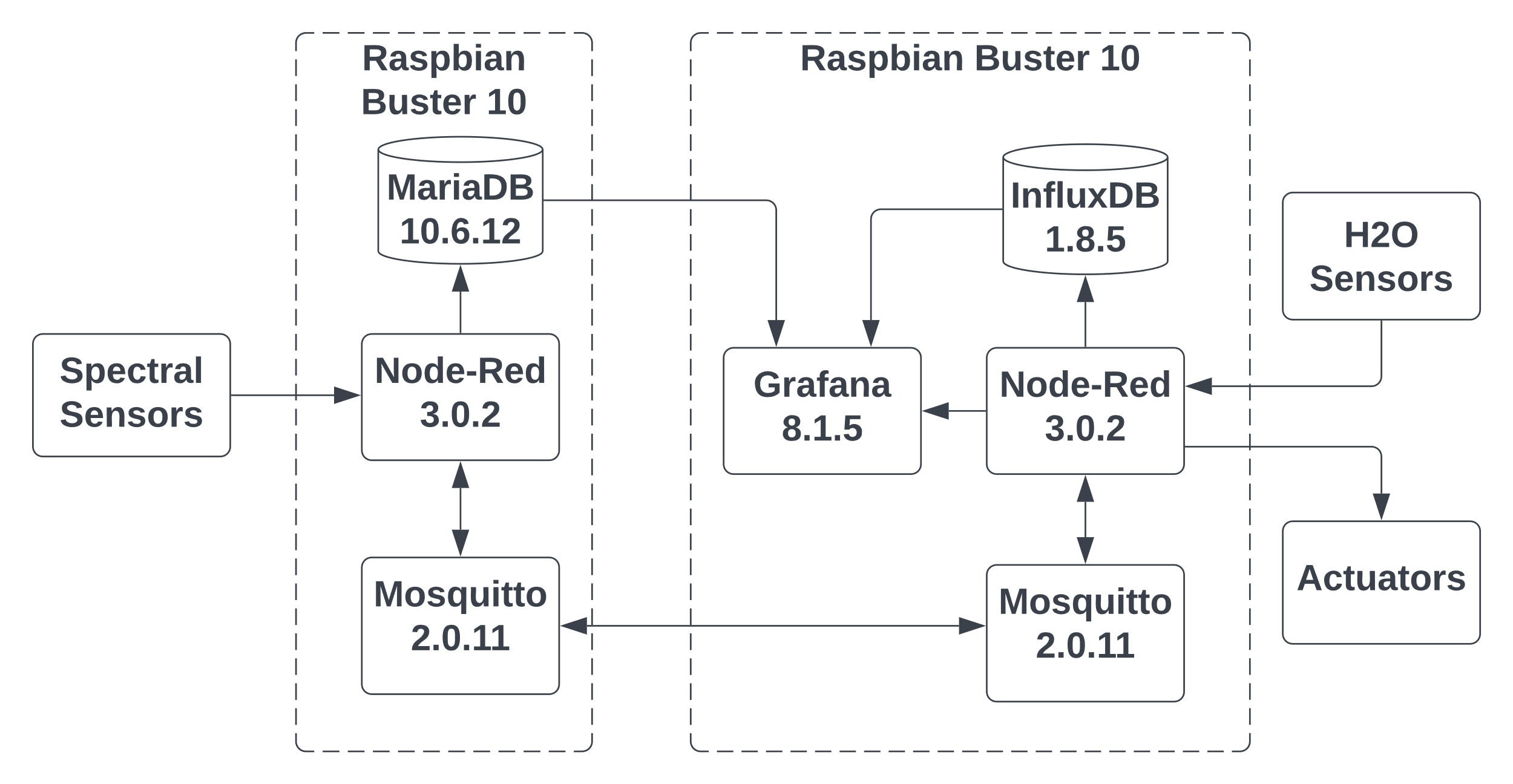


Figure 3 is a Data Flow Diagram that illustrates how the data is taken from the spectral sensors and combined with the user input to create a recommended quantity for nutrient adjustment and then stored for later analysis. The four AS7265x sensors are divided, two are placed in the control tank and two in the treatment tank. Each sensor produces 18 spectral channel readings. The system reads all 18 channels for each sensor and formats it into four separate JavaScript Object Notation (JSON) objects. The JSON objects are forwarded for aggregation with other system data and also to the nutrient prediction process. The nutrient prediction process computes the predicted ML content of the corresponding tank using a trained model and creates a JSON object. The predicted ML JSON is forwarded for aggregation and also sent to the ML comparer process for determining the difference between the current amount of nutrient and target nutrient level. The ML comparer compares the user input of the baseline ML, established through training the prediction model, and the current predicted ML and produces JSON object with the prescribed ML adjustment. Both predicted and prescribed ML are stored in the InfluxDB for the corresponding model and sensor. The user is required to manually input the daily EC for each tank, the baseline established in the model training for each sensor, and the amount of nutrient that was added to each tank when adjusting the ML. This data is read and stored into the respective local variable and is used in the aggregation process and determining the prescribed ML adjustment. The data aggregation process reads the JSON objects it receives and the variable values input by the user and combines all of data and writes it to the MariaDB with an SQL query.

Figure 3. Dataflow Diagram showing how the data moves in the system from the inputs to the storage.


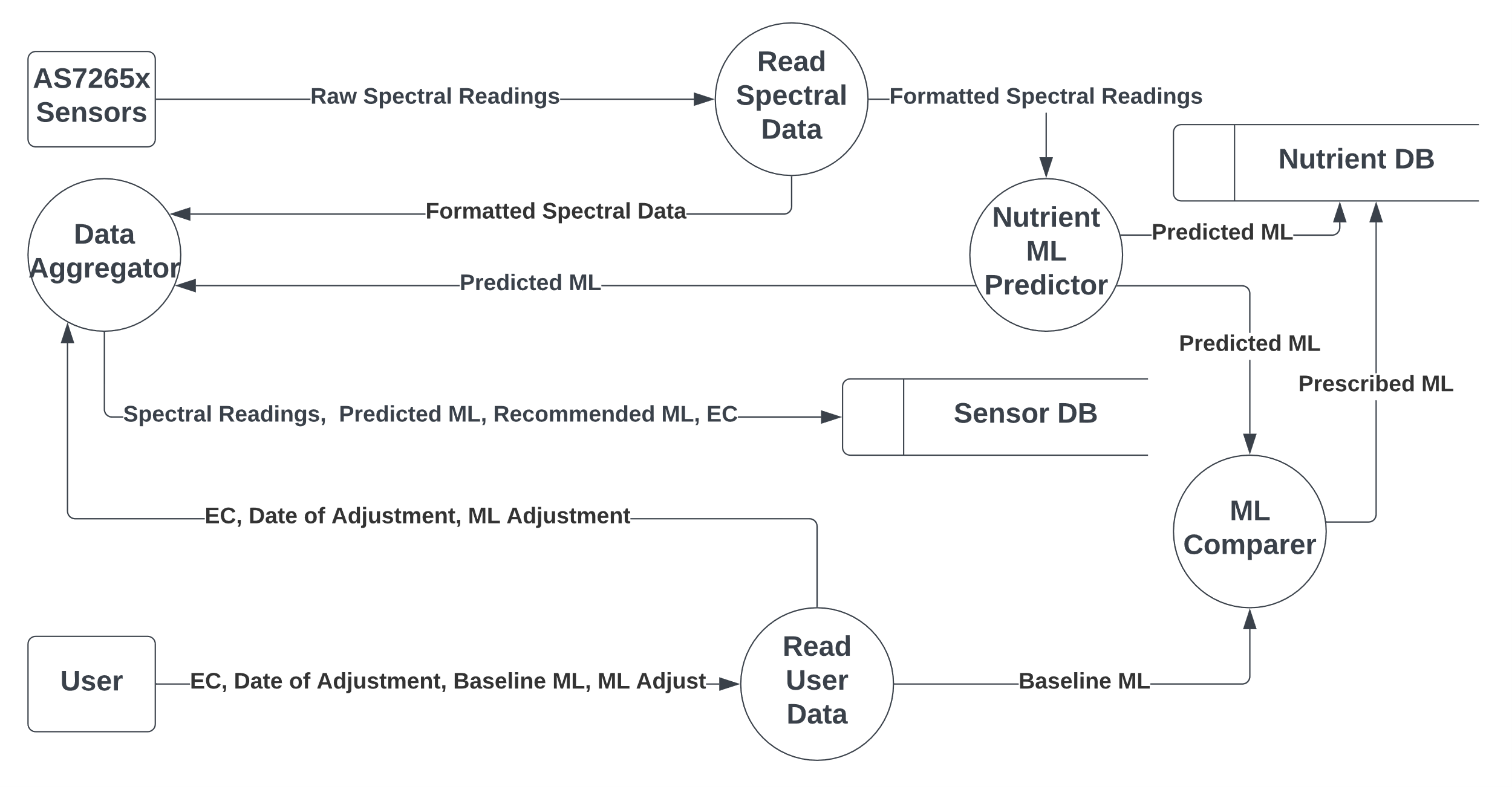


Finally, Figure 4 shows the systems algorithm used to produce the recommended nutrient ML quantity to add. First a test is done to check if 24 hours has passed since the last recommendation was given. If a full 24 hours has passed, a 22 hour mean is calculated of all the predicted values for day. Water is pumped to the flood tray for 20 minutes twice a day, during this time the water levels in the tank fall and the sensor readings are affected. Accounting for the empty tank period the daily mean excludes the two 60 minutes periods, starting from the time the pump switches on. Next a comparison of the current date to date of last adjustment is carried out. If the date is less than seven days, no recommendation is given. A seven-day buffer was used to eliminate any prediction anomaly and over adjustments of the nutrients. Another test is carried out to protect against model prediction anomalies.

The mean of the daily prediction is then compared to the baseline provided by the user. If the nutrient deficit is greater than 20 ML an adjustment is recommended in ML for the user to add. The user is then recommended to add the adjustment equal parts of Nutrient A and Nutrient B. Upon adjustment the user records the total sum quantity of nutrients added in ML and the date that the adjustment was made.

Figure 4. System algorithm used to recommend nutrient quantity to add.


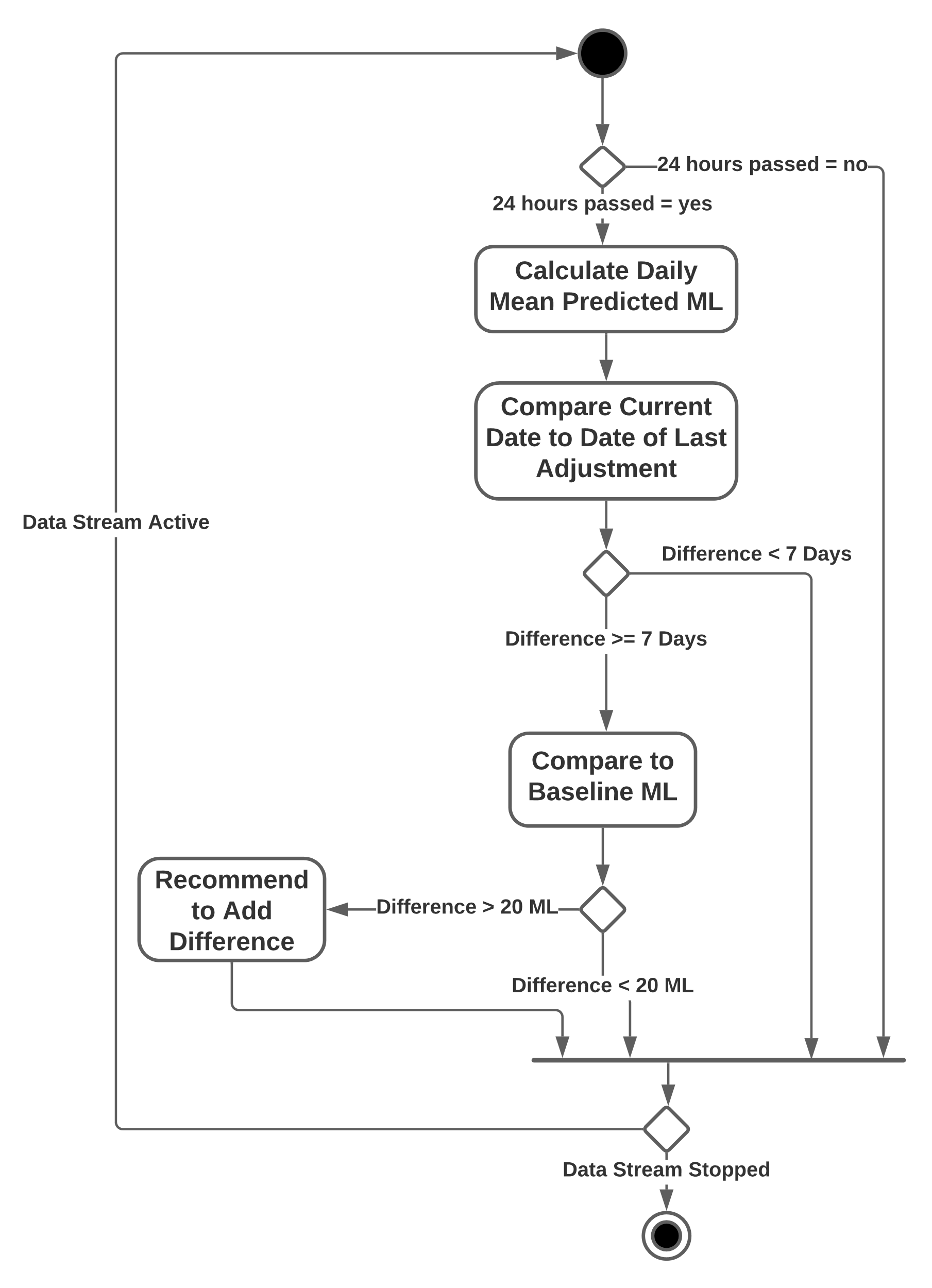

Supplement: S1 File — https://doi.org/10.6084/m9.figshare.25434514.v2. (DOCX) [file pone.0302638.s001.docx]
